# Contributions Made by CDC25 Phosphatases to Proliferation of Intestinal Epithelial Stem and Progenitor Cells

**DOI:** 10.1371/journal.pone.0015561

**Published:** 2011-01-25

**Authors:** Gwanghee Lee, Sofia Origanti, Lynn S. White, Jinwu Sun, Thaddeus S. Stappenbeck, Helen Piwnica-Worms

**Affiliations:** 1 Department of Cell Biology and Physiology, Washington University School of Medicine, St. Louis, Missouri, United States of America; 2 BRIGHT Institute, Washington University School of Medicine, St. Louis, Missouri, United States of America; 3 Molecular Imaging Center, Washington University School of Medicine, St. Louis, Missouri, United States of America; 4 Department of Pathology and Immunology, Washington University School of Medicine, St. Louis, Missouri, United States of America; 5 Department of Internal Medicine, Washington University School of Medicine, St. Louis, Missouri, United States of America; 6 Howard Hughes Medical Institute, Chevy Chase, Maryland, United States of America; Queensland University of Technology, Australia

## Abstract

The CDC25 protein phosphatases drive cell cycle advancement by activating cyclin-dependent protein kinases (CDKs). Humans and mice encode three family members denoted CDC25A, -B and -C and genes encoding these family members can be disrupted individually with minimal phenotypic consequences in adult mice. However, adult mice globally deleted for all three phosphatases die within one week after *Cdc25* disruption. A severe loss of absorptive villi due to a failure of crypt epithelial cells to proliferate was observed in the small intestines of these mice. Because the *Cdc25s* were globally deleted, the small intestinal phenotype and loss of animal viability could not be solely attributed to an intrinsic defect in the inability of small intestinal stem and progenitor cells to divide. Here, we report the consequences of deleting different combinations of *Cdc25s* specifically in intestinal epithelial cells. The phenotypes arising in these mice were then compared with those arising in mice globally deleted for the *Cdc25s* and in mice treated with irinotecan, a chemotherapeutic agent commonly used to treat colorectal cancer. We report that the phenotypes arising in mice globally deleted for the Cdc25s are due to the failure of small intestinal stem and progenitor cells to proliferate and that blocking cell division by inhibiting the cell cycle engine (through Cdc25 loss) versus by inducing DNA damage (via irinotecan) provokes a markedly different response of small intestinal epithelial cells. Finally, we demonstrate that CDC25A and CDC25B but not CDC25C compensate for each other to maintain the proliferative capacity of intestinal epithelial stem and progenitor cells.

## Introduction

The CDC25 phosphatases are critical components of the cell cycle engine that function to drive cell cycle transitions by dephosphorylating the CDKs [1]–[6]. We recently described a genetic model that enables the cell division cycle to be acutely halted in adult mice [7]. This was accomplished by the targeted disruption of genes encoding the CDC25 family of protein phosphatases. In this model, adult mice lacking two members of the CDC25 family (CDC25B and CDC25C) were globally deleted for the third family member (CDC25A) using transgenic mice expressing a tamoxifen-driven Cre recombinase from the ubiquitously expressed *Rosa26* (*R26*) locus [8]. Despite the fact that the CDC25s were deleted in all tissues of these triple knockout (*TKO*) mice, the major phenotype observed is in the small intestine where a loss of epithelial cell proliferation was accompanied by a corresponding loss of absorptive villi and animals died within a week after *Cdc25A* disruption. Despite the inhibition of cell division, overall crypt architecture was maintained and strikingly, neither apoptosis nor inflammation were observed to any significant level in the small intestines of these animals.

Given that the epithelium of the adult mammalian small intestine is in constant dialog with its underlying mesenchyme to direct progenitor proliferation, lineage commitment, terminal differentiation and ultimately cell death and given that Cre expression and therefore *Cdc25A* deletion occurred in cells of the underlying mesenchyme, the phenotypes observed in the small intestines of *TKO* mice could not be solely attributed to an intrinsic defect in the inability of small intestinal stem and progenitor cells to divide. For example, loss of CDC25s in neutrophils could have been responsible for the failure of inflammatory cells to infiltrate the small intestinal crypts of *TKO* mice. Therefore, we specifically deleted the CDC25s in intestinal epithelial cells by way of a tamoxifen-dependent Cre recombinase expressed from the murine villin promoter [9]. In addition, we generated mice that can be conditionally deleted for *CDC25B.* These mice were used to address several issues relevant to the CDC25 family of protein phosphatases and to the homeostasis of the small intestinal stem cell niche including (1) how the small intestine responds when cell division is blocked in epithelial cells but not in other cells and tissues of the small intestine such as mesenchyme, muscle, blood and endothelium; (2) how the small intestine responds to loss of epithelial cell proliferation induced by *Cdc25*-disruption versus by DNA damage (chemotherapy) (3) whether the failure to observe apoptosis and inflammation in the small intestines of *TKO* mice was due to the disruption of CDC25s in cells and tissues other than intestinal epithelial cells and (4) which of the three CDC25 family members are required to drive proliferation of intestinal epithelial stem cells and progenitors.

## Results

### Disruption of *Cdc25A* in intestinal epithelial cells of adult mice

**Figure 1:**
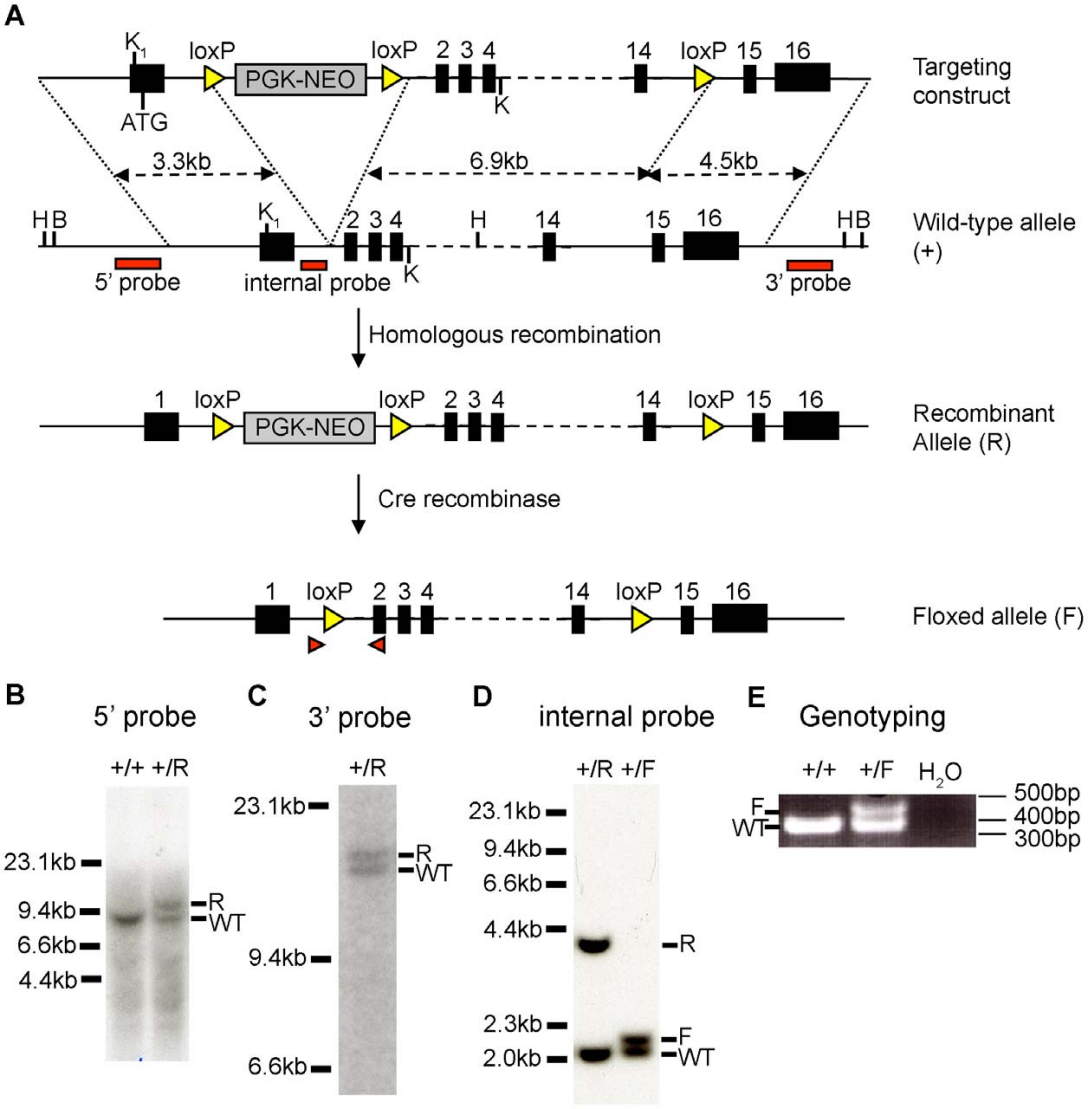
Targeted disruption of *Cdc25B* in mice. (**A**) Structure of targeting vector and chromosomal organization of *Cdc25B* locus before and after Cre-mediated excision. The genomic organization of the mouse *Cdc25B* gene was disrupted by inserting into intron 1 the neomycin phosphotransferase cDNA driven by the phosphoglycerine kinase promoter (pGK-neo) as a selectable marker. Exons are represented by black boxes. The location of Hind III (H), Bam HI (B) and Kpn I (K) site is indicated and *loxP* sites are represented by yellow triangles. Sizes of upstream (3.3 kb) and downstream (4.5 kb) homologous arms are indicated. Position of probes used for Southern blotting are shown. Red triangles depict the locations of PCR primers used for genotyping. Abbreviations: +, wild type allele; R, recombinant allele; F, floxed allele; WT, wild type. (**B**–**C**) Southern blot analysis demonstrating homologous recombination in the *Cdc25B* locus. ES cell genomic DNA was digested with Hind III (B) and Bam HI (C), and Southern blotting was performed using the 5′ and 3′ probes shown in panel A. The genotype of each ES cell line is indicated. The location of size markers is shown on the left. (**D**) Southern blot analysis demonstrating Cre-mediated recombination in the *Cdc25B* locus. ES cell clones containing the recombinant allele were expanded and transiently transfected with a plasmid encoding Cre recombinase. Genomic DNA was digested with Kpn I (K), and Southern blotting was performed using the internal probe shown in panel A. The genotype of each ES cell line is indicated. Location of size markers is shown on left. (**E**) PCR analysis of mouse tail DNA. Mouse tail DNA was amplified with PCR primers depicted as red triangles in panel A. The wild type (+) allele produced a 383 bp PCR product and floxed allele (F) produced a 433 bp PCR product. The genotype of each mouse is indicated. The location of size markers is shown on the right.

Mice expressing a tamoxifen-dependent Cre recombinase driven by the murine villin promoter (*vil-Cre-ER^T2^*) [9] were employed to disrupt *Cdc25s* specifically in intestinal epithelial cells. In these mice, administration of tamoxifen induces Cre-recombinase activity exclusively in small and large intestinal epithelial cells including stem cells. *vil-Cre-ER^T2^* mice were crossed with mice containing floxed alleles of *Cdc25A* [7] to generate mice containing one floxed and one null allele of *Cdc25A* (*vA^f/−^*). Additional crosses were carried out to generate mice containing one null and one floxed allele of *Cdc25A* that were also disrupted for *Cdc25B* (*vA^f/-^BKO*), *Cdc25C* (*vA^f/-^CKO*) or both *Cdc25B* and *Cdc25C* (*vA^f/-^BCKO*). In addition, mice containing floxed alleles of *Cdc25B* were generated and crossed with *vil-Cre-ER^T2^* transgenic mice togenerate mice with one null and one floxed allele of *Cdc25B* (*vB^f/−^*). The strategy used for generating *Cdc25B* conditional mice is shown in Fig. 1. Wild-type and *Vil-Cre-ER^T2^* mice were used as controls throughout our studies.

**Figure 2:**
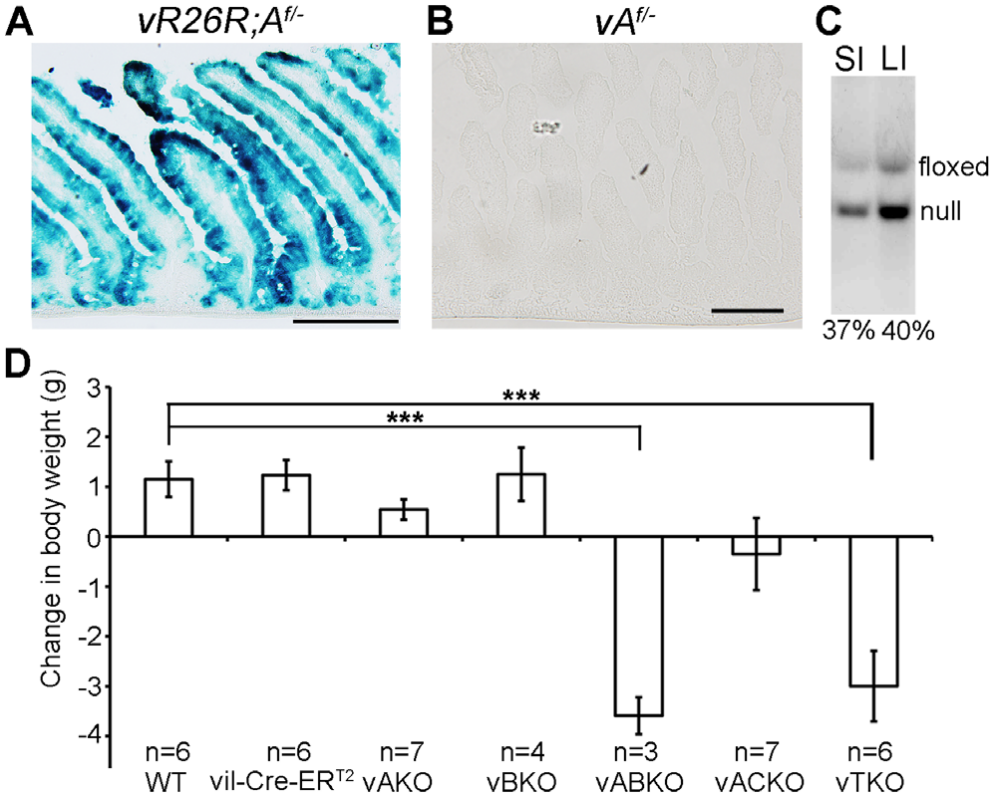
Specific deletion of *Cdc25A* in intestinal epithelial cells. (**A**, **B**) *vR26R*;*A^f/−^* and v*A^f/−^* mice were injected with tamoxifen for 5 consecutive days and then sacrificed 3 days after the final injection. Frozen sections of small intestines were prepared and stained with X-gal to visualize Cre-mediated deletion frequencies. Scale bar: 200 µm. (**C**) Genomic DNA isolated from the small and large intestines of *vA^f/−^* mice 3 days after the final tamoxifen-injection was digested with Bst XI followed by Southern blotting. Deletion frequencies are shown below the each lane. (**D**) Mice were weighed prior to injection and 3 days after the final tamoxifen injection. Data is presented as mean +/− standard error of the mean (SEM). Asterisks indicate significantly different from WT mice injected with tamoxifen as determined by a Student's t-test. *, P<0.05; **, P<0.01; ***, P<0.001. The actual P-values are 0.86 (*vil-Cre-ER^T2^*), 0.15 (*vAKO*), 0.87 (*vBKO*), 0.00007 (*vABKO*), 0.11(*vACKO*) and 0.0004 (*vTKO*).

*vA^f/-^* mice were bred to a Cre-inducible β-galactosidase reporter line (*R26R*) [10] to enable deletion efficiencies at floxed loci to be readily monitored. X-gal (5-bromo-4-chloro-3-indolyl-b-D-galactopyranoside) staining revealed efficient recombination when tamoxifen was dosed at a concentration of 4 mg per 25 g body weight for 5 consecutive days (Fig. 2A, B). X-gal staining was observed exclusively in the epithelium of both the small and large intestines demonstrating the specificity of the villin promoter for these tissues (data not shown). The gross and histological appearance of all organs, with the exception of intestines (see below), was indistinguishable between tamoxifen-injected *vA^f/-^*BKO and *vA^f/-^BCKO* and all other mice. The deletion frequency (conversion of floxed to null allele) was ∼40% in the small and large intestines (Fig. 2C). However, this is likely an underestimate given the presence of DNA derived from non-epithelial cell types (muscle, blood and mesenchyme) that do not express Cre recombinase.

### Significant shortening of small intestines in v*ABKO* and v*TKO* mice

Mice globally deleted for all three CDC25s exhibit an ∼20% reduction in body weight within one week of the induction of global Cre expression [7]. This weight loss was also associated with significant mortality. To determine the consequences of deleting *Cdc25A* specifically in intestinal epithelium (in a background of complete knockouts for *Cdc25B* and *Cdc25C*), the weights of *vTKO* mice were compared prior to injection (day 1) and prior to sacrifice on day 8 (Fig. 2D). A significant decrease in weight (17%) and increased mortality (5 of 11 *vTKO* mice died by day 8) were also observed in these mice. Interestingly, a similar weight loss was observed when only two family members (*Cdc25A* and *Cdc25B*) were disrupted in intestinal epithelium (*vABKO* mice) and *vABKO* mice also showed increased mortality by day 8 (2 of 5 *vABKO* mice died by day 8). In contrast, mice disrupted for *Cdc25A* alone, *Cdc25B* alone, or both *Cdc25A* and *Cdc25C* were not significantly different from control littermates (WT and *vil-Cre-ER^T2^*; Fig. 2D). These results indicate that small intestinal homeostasis in mice requires CDC25A or CDC25B but not CDC25C.

**Figure 3:**
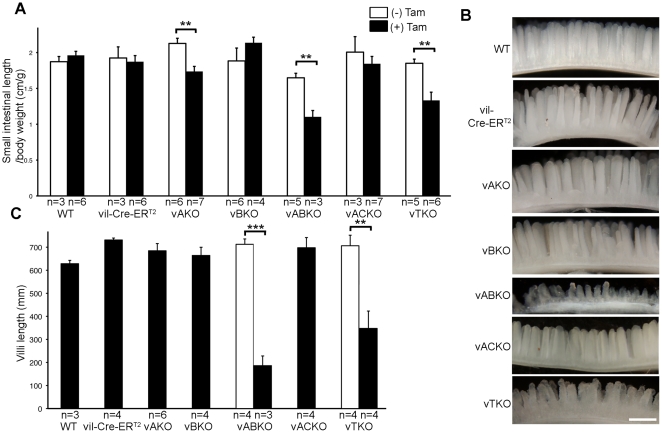
Loss of homeostasis in small intestines of *vABKO a*nd *vTKO* mice. (**A**) Mice were injected with tamoxifen for five consecutive days and then sacrificed 3 days after the final injection. Small intestines were isolated and length determinations were made. Small intestine lengths were normalized to body weights, which were determined prior to the first tamoxifen-injection. Data is presented as mean +/− SEM. Asterisk (*) indicates significantly different after tamoxifen injection as determined by a Student's t-test. *, P<0.05; **, P<0.01; ***, P<0.001. The actual P-values are 0.38 (WT), 0.76 (*vil-Cre-ER^T2^*), 0.003 (*vAKO*), 0.31 (*vBKO*), 0.002 (*vABKO*), 0.46 (*vACKO*) and 0.004 (*vTKO*). The small intestinal lengths of *vAKO*, *vABKO* and *vTKO* mice were significantly different from WT mice injected with tamoxifen as determined by a Student's t-test. *, P<0.05; **, P<0.01; ***, P<0.001. Actual P-values are 0.40 (*vil-Cre-ER^T2^*), 0.03 (*vAKO*), 0.10 (*vBKO*), 0.00006 (*vABKO*), 0.36 (*vACKO*) and 0.0006 (*vTKO*). (**B**) Duodenums isolated from mice treated as described in A were photographed under a dissection microscope. Scale bar: 0.5 mm. (**C**) Significant shortening of villi in small intestines of *vABKO* and *vTKO* mice. Length of individual villi shown in panel B were measured (30 villi per mouse). Data is presented as mean +/− SEM. Asterisk (*) indicates significantly different after tamoxifen injection as determined by a Student's t-test. P-values are 0.00008 (*vABKO*) and 0.007 (*vTKO*). Villi lengths of *vil-Cre-ER^T2^*, *vABKO* and *vTKO* mice were significantly different from WT mice injected with tamoxifen as determined by a Student's t-test. *, P<0.05; **, P<0.01; ***, P<0.001. Actual P-values are 0.001 (*vil-Cre-ER^T2^*), 0.27 (*vAKO*), 0.45 (*vBKO*), 0.0005 (*vABKO*), 0.25 (*ACKO*) and 0.03 (*vTKO*).

**Figure 4:**
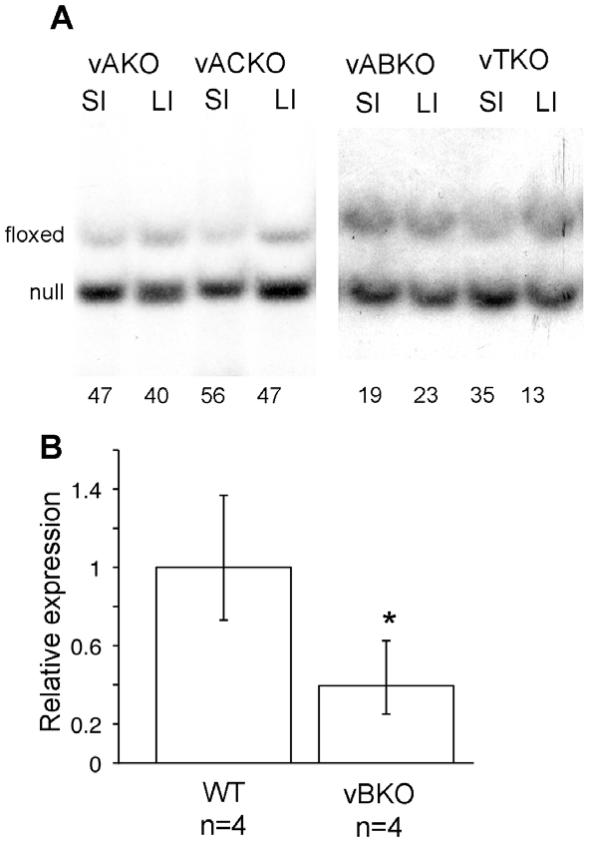
Tamoxifen injection induced efficient recombination within *Cdc25A* and *Cdc25B* loci. (**A**) Genomic DNA isolated from the small and large intestines of tamoxifen-treated mice were assessed for Cre-mediated excision by Southern blotting. Deletion frequencies are shown below each lane and were determined by measuring band intensities using a Molecular Dynamics Storm imager. (B) Total RNA isolated from the small intestine (jejunum) of tamoxifen-treated WT and *vB^f/−^* mice was reverse-transcribed into cDNA. qRT-PCR was used to determine relative amounts of *Cdc25B* mRNA. The data is presented as mean +/− SEM. Asterisk (*) indicates significantly different from WT, P  =  0.012 by Student's t-test. Note that *vB^f/−^* mice are generated by crossing *Cdc25B* null mice [26] with *Cdc25B* conditional mice. The PCR primers detect transcript arising from the null allele but not the deleted floxed allele. Thus, a 50% decrease in relative expression indicates complete loss of *Cdc25B* expression.

**Figure 5:**
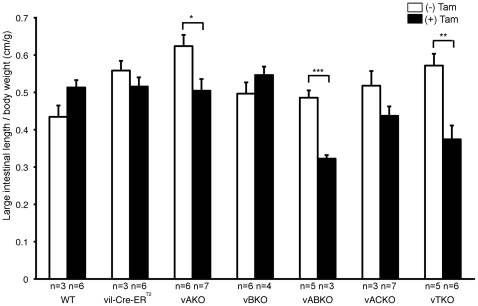
Significant shortening of large intestine of *vABKO* and *vTKO* mice. Mice were injected with tamoxifen for five consecutive days and then sacrificed 3 days after the final injection. Large intestines were isolated and length determinations were made. Large intestine lengths were normalized to body weights, which were determined prior to the first tamoxifen-injection. Data is presented as mean +/− SEM. Asterisks indicate significantly different after tamoxifen injection as determined by a Student's t-test. P-values are 0.059 (WT), 0.32 (*vil-Cre-ER^T2^*), 0.020 (*vAKO*), 0.27 (*vBKO*), 0.0009 (*vABKO*), 0.12 (*vACKO*) and 0.003 (*vTKO*). Large intestinal lengths of *vABKO*, *vACKO* and *vTKO* mice were significantly different from WT mice injected with tamoxifen as determined by a Student's t-test. P-values are 0.94 (*vil-Cre-ER^T2^*), 0.82 (*vAKO*), 0.30 (*vBKO*), 0.00034 (*vABKO*), 0.041 (*vACKO*) and 0.0077 (*vTKO*).

A significant shortening of the small intestines was measured in *vABKO* (44%) and *vTKO* (32%) mice relative to WT mice within one week of the first tamoxifen injection (Fig. 3A). This phenotype was not a condition existing prior to tamoxifen injection, as small intestinal lengths of *vA^f/−^BKO and vA^f/−^BCKO* mice were not different from controls (Fig. 3A). In contrast, the length of the small intestine did not change significantly after tamoxifen injection of *vB^f/−^* or *vA^f/−^CKO* animals (Fig. 3A). A mild decrease (∼11%) in small intestinal length was observed in animals conditionally deleted for *Cdc25A* (*vAKO*). As additional controls, wild type littermates (WT) and *vil-Cre-ER^T2^* transgenic mice were also injected with tamoxifen and showed no significant differences in small intestinal length (Fig. 3A). We noted that the frequency of Cre-mediated deletion of the floxed *Cdc25A* allele in the small intestines of tamoxifen-treated mice was comparable for each genotype (compare Fig. 2C and Fig. 4). Since the villin promoter expresses Cre in both small and large intestinal epithelium, we also measured the lengths of the large intestines of mice before and after tamoxifen treatment. Lengths of large intestines were significantly shorter in both *vABKO* (37% decrease) and *vTKO* (27% decrease) mice (Fig. 5). Mice doubly deleted for both *Cdc25A* and *Cdc25C* (*vACKO*) also showed a modest (15%) but significant (p  =  0.04) reduction in large intestinal length.

### Small intestinal phenotypes in tamoxifen-treated mice

Disruption of both *Cdc25A* and *Cdc25B* (*vABKO*) or all three *Cdc25s* (*vTKO*) in intestinal epithelium also caused a significant loss of small intestinal villus height (duodenum is shown in Fig. 3B with corresponding quantification in Fig. 3C). Shortened villus height was also observed in the jejunums and ileums of these mice (data not shown). In contrast, disruption of a single *Cdc25* family member or both *Cdc25A* and *Cdc25C* in intestinal epithelium did not affect small intestinal villus height. Taken together, these results demonstrate that loss of *Cdc25C* does not contribute to the phenotypes observed in *TKO* mice. Furthermore, the decrease in villus height measured in tamoxifen-treated *vA^f/−^BCKO* mice (45%) was similar to that observed in mice globally deleted for all three *Cdc25s* (47%) [7]. These results argue that the phenotypes observed in the small intestines of mice globally deleted for all three *Cdc25*s is due to defects in intestinal epithelial cells and that loss of the *Cdc25s* in neighboring mesenchyme does not contribute to the observed phenotypes.

**Figure 6:**
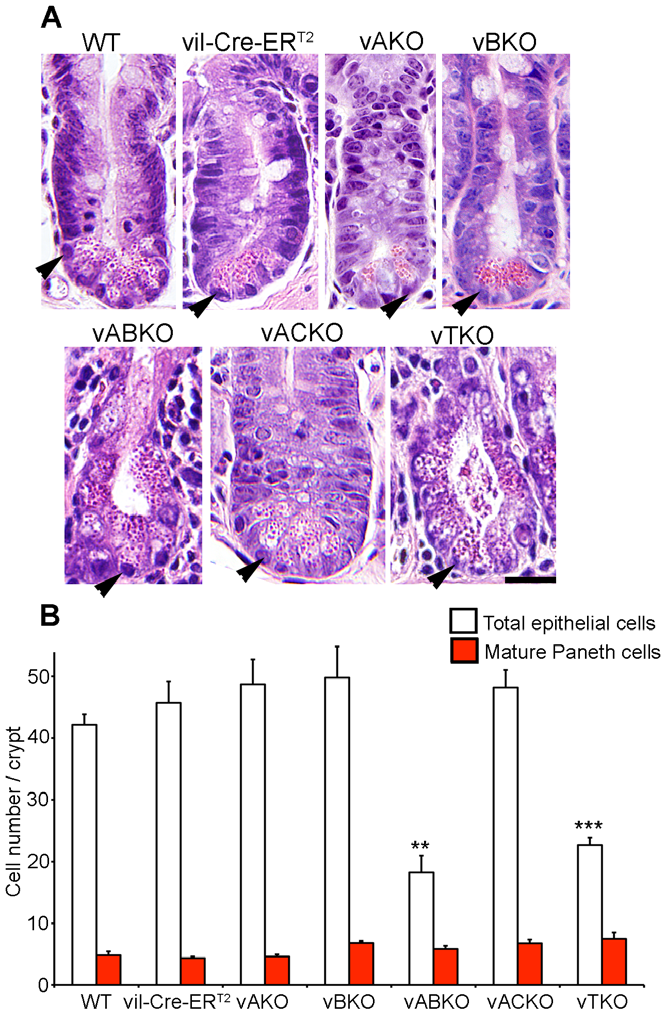
Response of crypt epithelial cells to loss of CDC25s. (**A**) Mice were treated with tamoxifen for five consecutive days and sacrificed 3 days after the final injection. Small intestines were isolated and fixed. 5 µm tissue sections were prepared and stained with Hematoxylin and Eosin. Arrow heads indicate Paneth cells. Scale bar: 0.5 mm. (**B**) Crypts within the proximal portion of the small intestine of tamoxifen treated mice were examined for epithelial cells and Paneth cells. Areas containing Brunner's gland were excluded from analysis. Twenty crypts were counted per mouse and three mice of each genotype were evaluated. Similar patterns were observed in mid and distal portions of the small intestine (data not shown). Data is presented as mean +/− SEM. Asterisk (*) indicates significantly different from WT mice as determined by a Student's t-test (total epithelial cells). *, P<0.05; **, P<0.01; ***, P<0.001. Actual P-values are 0.41 (*vil-Cre-ER^T2^*), 0.21 (*vAKO*), 0.23 (*vBKO*), 0.002 (*vABKO*), 0.15 (*ACKO*) and 0.0007 (*vTKO*). *vBKO* mice have slightly more mature Paneth cells per crypt compared to WT mice as determined by a Student's t-test. P-values are 0.52 (*vil-Cre-ER^T2^*), 0.77 (*vAKO*), 0.044 (*vBKO*), 0.28 (*vABKO*), 0.076 (*ACKO*) and 0.098 (*vTKO*).

Histological examination of small intestines also revealed loss of epithelial cells within crypts of *vABKO* and *vTKO* mice (Fig. 6A). Cellular composition of crypts was quantified by counting the number of crypt epithelial cells and Paneth cells (Fig. 6B). The number of epithelial cells/crypt was greatly diminished in *vABKO* (57% decrease) and *vTKO* (46% decrease) mice. Despite the reduced cellularity of crypts in these mice, individual epithelial cells appeared to undergo a process of hypertrophy, thus accounting for the maintenance of overall crypt structure observed in *vABKO* and *vTKO* mice.

**Figure 7:**
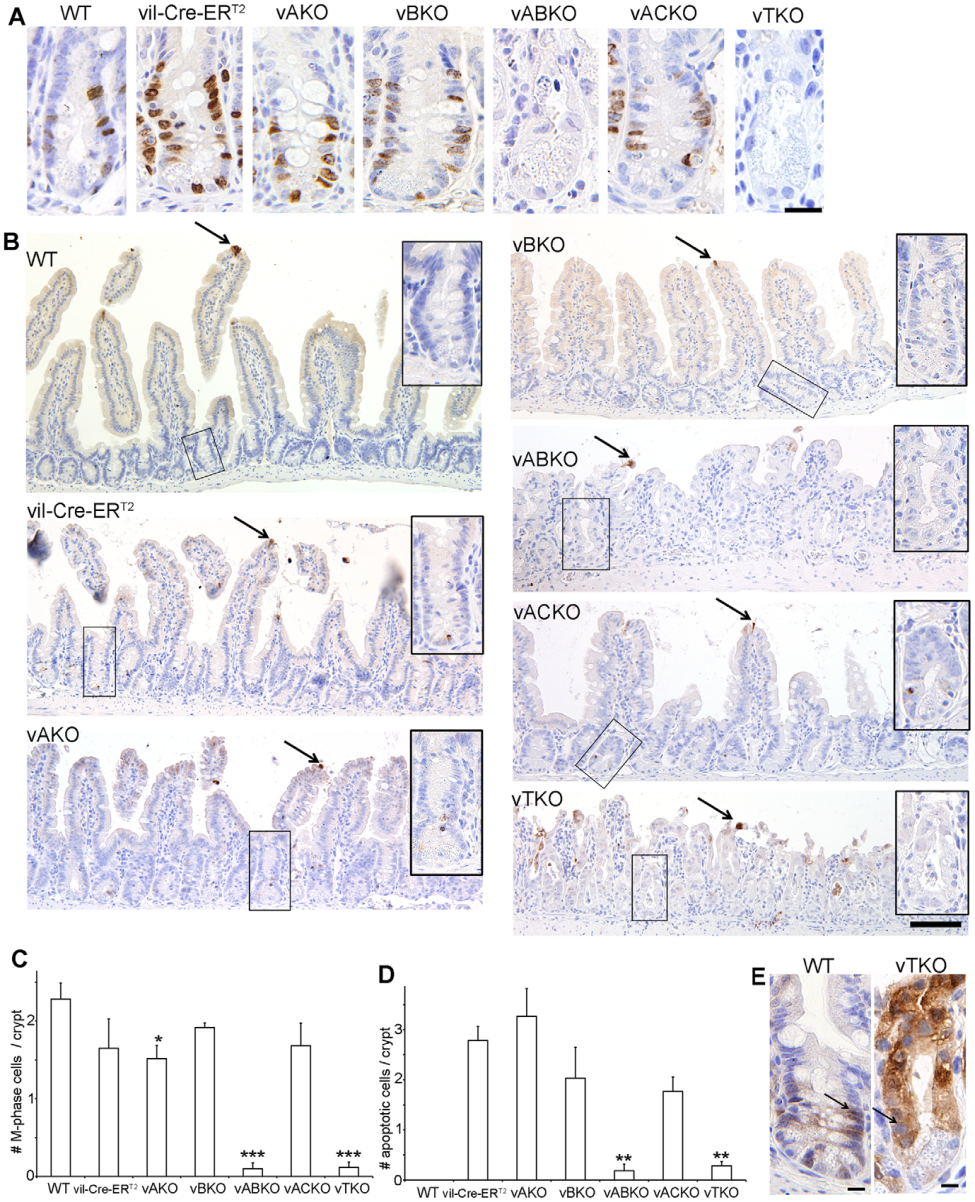
Proliferation and apoptosis in crypts of *Cdc25*-disrupted mice. (**A**) Mice were injected with tamoxifen for five consecutive days and then sacrificed 3 days after the final injection. One hour prior to sacrifice, mice were injected with BrdU. Intestines were isolated, and sections were stained with BrdU antibody (brown) and counterstained with hematoxylin (blue). Scale bar: 20 mm. (**B**) Mice were injected with tamoxifen for five consecutive days and were sacrificed 3 days after last tamoxifen injection. Intestines were isolated and sections were stained for cleaved caspase-3. 3, 3′-diaminobenzidine (DAB, brown) was used as a substrate, and sections were counter-stained with hematoxylin. Arrows indicate cells at the tip of villi, which stain positive for cleaved caspase-3. Scale bar: 0.1 mm. (**C–D**) Crypts within the proximal portion of the small intestine of tamoxifen treated mice were examined for mitotic cells (presence of mitotic figures) (C) and apoptotic cells (presence of fragmented nuclei) (D). Areas containing Brunner's gland were excluded from analysis. Twenty crypts were counted per mouse and three mice of each genotype were evaluated. Similar patterns were observed in mid and distal portions of the small intestine (data not shown). Data is presented as mean +/− SEM. Asterisks in panel C indicate significantly different from WT mice as determined by a Student's t-test (M-phase cells). *, P<0.05; **, P<0.01; ***, P<0.001. Actual P-values are 0.22 (*vil-Cre-ER^T2^*), 0.045 (*vAKO*), 0.16 (*vBKO*), 0.00056 (*vABKO*), 0.17 (*ACKO*) and 0.00055 (*vTKO*). Asterisks in panel D indicate significantly different number of apoptotic cells from *WT* mice as determined by a Student's t-test. P-values are 0.48 (*vAKO*), 0.33 (*vBKO*), 0.0011 (*vABKO*), 0.064 (*ACKO*) and 0.0010 (*vTKO*). (**E**) Mice were injected with tamoxifen as described in A and small intestines were isolated, sectioned and stained for inactive Cdk1 (phosphorylated on Tyr-15, brown) and counterstained with hematoxylin (blue). Arrows indicate epithelial cells positively stained with the phospho-Tyr 15 CDK1 antibody. Scale bar: 20 µm.

The number of mature Paneth cells/crypt was similar in *vABKO* and *vTKO* mice relative to controls suggesting that loss of CDC25s impacted replicating progenitors rather than fully differentiated Paneth cells (Fig. 6B). Indeed, mitotic cells were nearly absent in *vABKO* and *vTKO* mice (Fig. 7C). In addition, BrdU-labeling experiments revealed a significant decrease of S-phase cells in the crypts of *vABKO* and *vTKO* mice (Fig. 7A). We reported previously that intestinal epithelial cells arrest in the G1- and G2- phases of the cell division cycle upon global deletion of the CDC25s (17). The CDKs are regulated by reversible phosphorylation and phosphorylation of tyrosine 15 (phospho-Tyr-15) maintains both CDK1 and CDK2 in an inactive state until this residue is dephosphorylated by the CDC25s. We predicted that levels of phospho-Tyr-15 would be elevated in crypt epithelial cells lacking the CDC25s. As seen in Fig. 7E, levels of phospho-Tyr-15 Cdk1 are greatly elevated in the crypts of *vTKO* mice compared with *WT* mice. This observation is consistent with the loss of S- (Fig. 7A) and M-phase (Fig. 7C) cells in *vTKO* mice.

Cell death did not appear to play a role in the crypt epithelial cell loss of *vABKO* and *vTKO* mice as only occasional apoptotic cells (∼1 apoptotic cell per 4 or 5 crypts) were observed in the crypts of these mice (Fig. 7B and 7D). In contrast, a higher level of apoptosis (up to 6% of total epithelial cells/crypt) was observed in tamoxifen-treated *vil-Cre-ER^T2^*, *vA^f/−^*, *vB^f/−^* and *vA^f/−^CKO* mice. Apoptosis was not observed in tamoxifen-treated WT mice. These results indicate that apoptosis is caused by Cre expression in proliferating epithelium. This conclusion is supported by the observed low level of apoptosis in small intestinal crypts of *vABKO* and *vTKO* mice where proliferation was halted (Fig. 7A).

### Enhanced Wnt signaling and differentiation in intestinal epithelial cells disrupted for *Cdc25* family members

**Figure 8:**
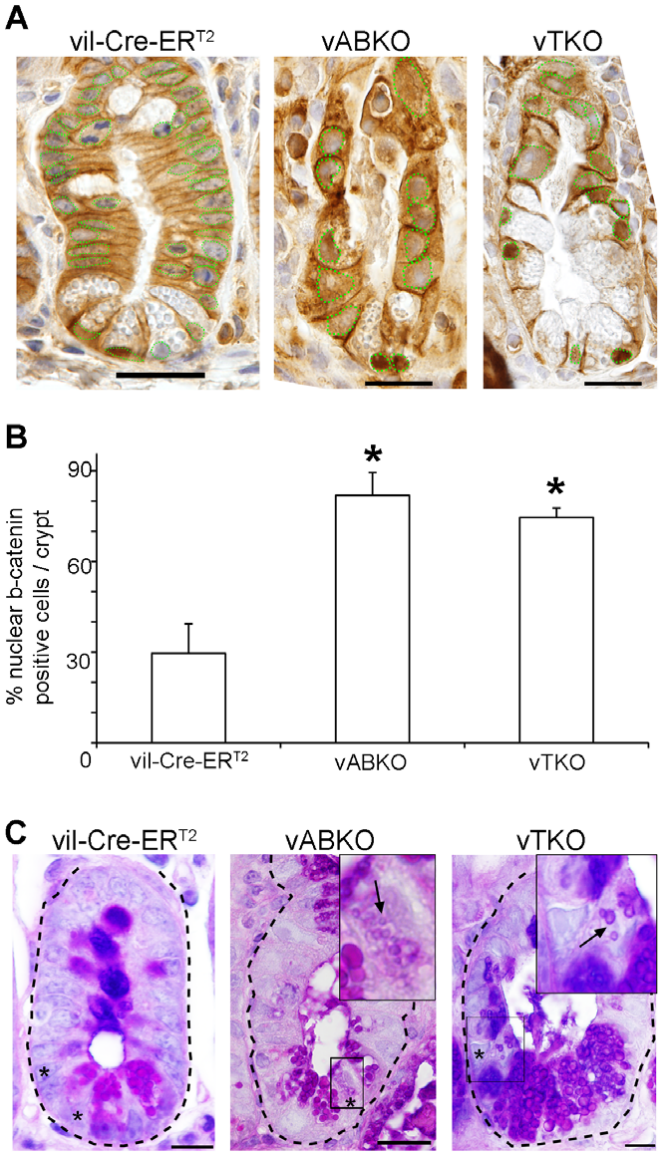
Enhanced Wnt-signaling and differentiation of CBC cells in *vABKO* and *vTKO* mice. (**A**) Tissue sections of small intestines prepared from *Vil-Cre-ER^T2^* (left panel), *vABKO* (middle panel) and *vTKO* (right panel) mice were stained with antibodies specific for beta-catenin (green). Nuclei were stained with hematoxylin (blue). Green hatched lines outline nuclei. Scale bar: 20 µm. (**B**) Quantitation of nuclear beta-catenin staining is shown in A. Data is presented as mean +/− SEM. Asterisks in panel B indicate significantly different from vil-Cre-ER^T2^ mice as determined by a Student's t-test. (**C**) Intestinal sections were stained with Periodic Acid Schiff/alcian blue to label Paneth cells and goblet cells from *Vil-Cre-ER^T2^* (left panel), *vABKO* (middle panel) and *vTKO* (right panel) mice. Insets are magnifications of boxed regions shown in middle and right panel. Asterisks in left panel indicate CBC cells. Asterisks in middle and right panels indicate immature Paneth cells. Arrows in middle and right panel indicate small granules of immature Paneth cells. Scale bar: 10 µm.

Acute disruption of cell proliferation in small intestinal crypts, due to global CDC25 loss, leads to enhanced Wnt-signaling and concomitant differentiation of small intestinal epithelial cell progenitors along multiple lineages [7]. Thus, the presence of nuclear beta-catenin was monitored to determine if Wnt signaling was also enhanced when *Cdc25s* were specifically disrupted in intestinal epithelium. As seen in Fig. 8(A, B) significantly more epithelial cells stained positive for nuclear β-catenin in both *vABKO* (middle panel) and *vTKO* (right panel) mice compared with those in *vil-Cre-ER^T2^* (left panel) mice.

Canonical Wnt-signaling has many functions in the small intestines including maintaining crypt structure [11], [12], promoting differentiation of progenitors along the secretory lineage [13]–[15] and maintaining the self-renewal capacity of stem cells [16]–[18]. Histologic sections of *vABKO* and *vTKO* small intestinal crypts also showed extensive premature differentiation along multiple epithelial lineages. PAS/alcian blue-stained sections of *vABKO* and *vTKO* small intestines did not reveal Crypt Base Columnar (CBC) cells. Instead, cells with scattered, small PAS/alcian blue-positive apical granules were observed, consistent with the conclusion that CBC cells differentiated along the Paneth cell lineage under these conditions (Fig. 8C).

**Figure 9:**
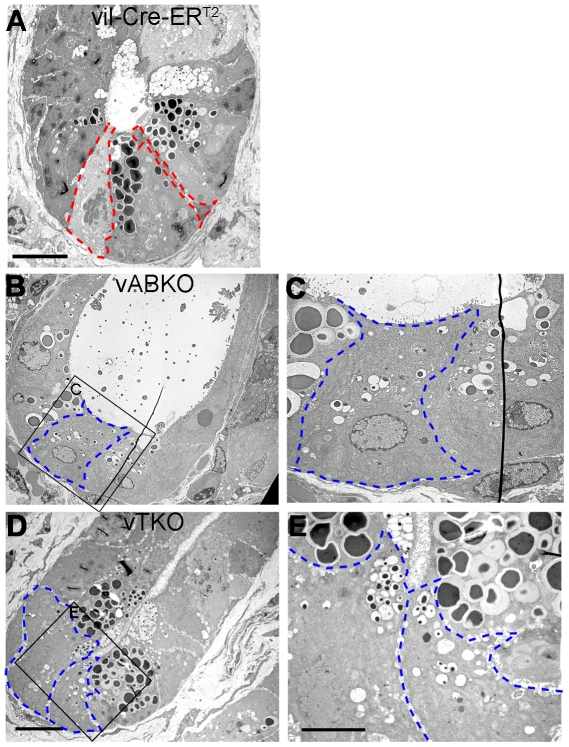
Differentiation of CBC cells into immature Paneth Cells in *Cdc25*-disrupted mice. Small intestines were dissected and processed for Transmission Electron Microscopy. EM sections of crypts from *vil-Cre-ER^T2^* (A), *vABKO* (B, C) and *vTKO* (D, E) mice. Insets in panels B and D are shown at higher magnification in panels C and E, respectively. The red-hatched lines in panel A demark two CBC cells separated by a mature Paneth cell. Blue-hatched lines in panels B-D outline borders of immature Paneth cells arising from premature differentiation of CBC cells. Note that differentiating CBC cells also undergo hypertrophy. Scale bar: 10 µm (A, B, D), 5 µm (C, E).

Electron microscopic analysis confirmed the effects of epithelial cell cycle arrest on differentiation (Fig. 9). Both mature and immature Paneth cells (the latter contains much smaller granules than mature cells) were present in crypt bases of *vABKO* (Fig. 9B, C) and *vTKO* (Fig. 9D, E). CBC cells were not readily identified in crypts of these mice and were replaced instead by immature Paneth cells. These immature Paneth cells were not readily apparent in H& E stained sections due to the small size of granules (Fig. 6) but were readily observed upon staining with Periodic Acid Schiff/alcian blue (Fig. 8C) or by EM (Fig. 9). Taken together, these results confirm the observation that loss of epithelial cell proliferation causes premature differentiation of CBC cells into Paneth cells.

### Response of intestinal epithelial cells to chemotherapy versus *Cdc25* deletion

Blocking CDC25 function directly inhibits cell division by preventing activation of the cell cycle engine. In contrast, radiation and chemotherapy, current mainstays of cancer treatment, inhibit cell division indirectly by inducing DNA damage. Our hypothesis was that the effects of directly blocking cell cycle would be less damaging to the intestinal mucosa. Therefore, we directly compared the intestinal mucosa of mice with CDC25 inactivation to mice treated with the topoisomerase I inhibitor irinotecan, which induces DNA damage thereby leading to cell cycle arrest [19]. Irinotecan was chosen for this comparison as it is clinically effective against several human cancers, most notably colorectal cancer [20], [21]. However, mucositis is a frequent side effect of this drug [22].

**Figure 10:**
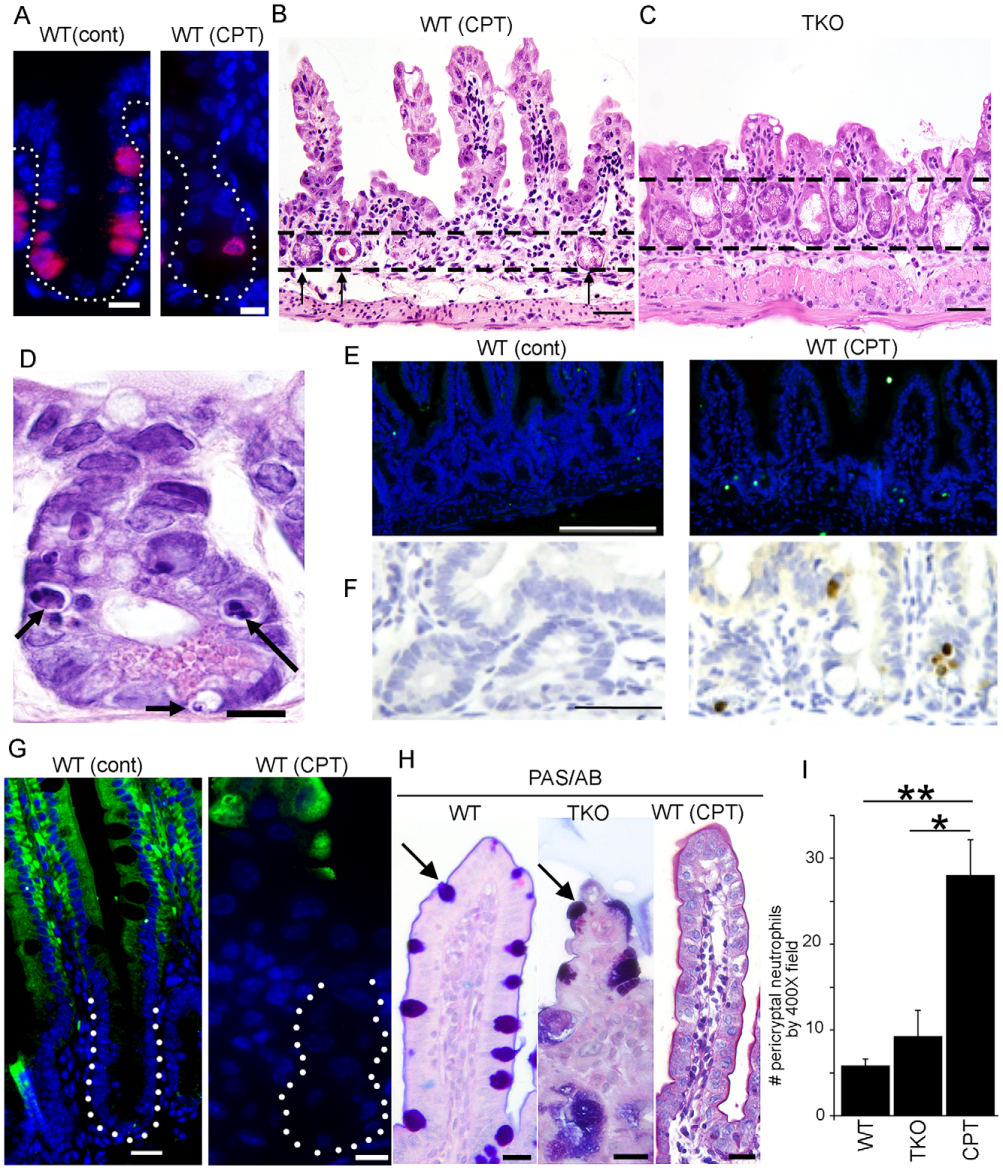
Response of small intestinal epithelial cells to irinotecan. (**A**) Significant decrease in the number of BrdU postive cells in the small intestinal crypts of irinotecan-treated mice. WT mice were injected with PBS (cont) or irinotecan (CPT) for 6 consecutive days and sacrificed on day 7. One hour prior to sacrifice, mice were injected with BrdU. Intestines were isolated and sections were stained with an antibody specific for BrdU. Dotted lines demark crypt margins. Red: BrdU, Blue: nuclei (DAPI). Scale bar: 10 µm. (**B**, **C**) Significant loss of crypts in the small intestines of irinotecan-treated mice. Intestines were isolated from irinotecan-treated mice (B) and *TKO* mice (C), and sections were stained with H & E. Arrows in (B) indicate remaining crypts. Scale bar: 50 µm. (**D–F**) Irinotecan induces significant apoptosis in the crypts of treated mice at early time points after irinotecan-treatment. (D) Intestinal sections were prepared after two irinotecan administrations and stained with H & E. Arrows depict apoptotic cells. Scale bar: 10 µm. (E) Intestinal sections from WT mice treated with PBS (cont)) and irinotecan (CPT) were stained by terminal deoxynucleotidyl transferase dUTP nick end labeling (TUNEL) assay. Green: TUNEL, Blue: nuclei (DAPI). Scale bar: 100 µm. (F) Intestinal sections from WT mice treated with PBS (left) and irinotecan (right) were stained for cleaved caspase-3. DAB (3, 3′-diaminobenzidine) was used as a substrate (brown) and sections were counter-stained with hematoxylin (blue). Scale bar: 50 µm. (**G**) Differentiation along the enterocyte lineage is not affected in irinotecan-treated mice. Intestines isolated from PBS- or irinotecan-treated WT mice were stained with the enterocyte marker, L-Fabp. Dotted lines demark crypt margins. Green: L-Fabp, Blue: nuclei (DAPI). Scale bar: 10 µm. (**H**) Loss of goblet cells in irinotecan-treated mice. Intestinal sections were stained with Periodic Acid Schiff/alcian blue to label goblet cells (indicated with arrow). Sections from WT (left), *TKO* (center) and irinotecan-treated WT mouse (right) are shown. Scale bar: 20 µm. (**I**) Significant infiltration of neutrophils in crypts of irinotecan-treated mice. The numbers of neutrophils were counted in 10 randomly chosen microscopic fields at 40X magnification in untreated WT, *TKO* and irinotecan-treated WT mice (CPT). Asterisks indicate significantly different from WT as determined by Student's t-test. WT and *TKO*: not significant, P = 0.33; WT and CPT: significant, P = 0.0006; *TKO* and CPT: significant, P = 0.02.

A profound loss of proliferation was observed in the small intestines of irinotecan-treated mice (Fig. 10A). The intestinal mucosa in irinotecan-treated mice showed multiple features of damage that were quite distinct from the mucosa of *TKO* mice: i) loss of crypts (Fig. 10B), ii) increased apoptosis in the crypts (Fig. 10 (D,E,F)), iii) lack of premature enterocytic differentiation in the crypts (Fig. 10G), iv) loss of villus goblet cells (Fig. 10H), and v) increase in acute inflammatory cells (Fig. 10I). Taken together these findings demonstrate that directly blocking the cell division through CDC25 loss is less damaging to the intestinal mucosa than is blocking cell division by inducing DNA damage with chemotherapy. Note that inflammation was not observed in *TKO* mice where CDC25 loss theoretically occurs in all cells and tissues of the mouse (including inflammatory cells) or in *vTKO* mice where CDC25 loss is restricted to intestinal epithelium (Fig. 10I and data not shown). Thus, failure to observe inflammation in TKO mice cannot be attributed to an intrinsic proliferative defect in the inflammatory cells themselves.

## Discussion

Here we describe a genetic model that enables the cell division cycle to be acutely halted in the intestinal epithelium of adult mice. This was accomplished by the targeted disruption of the CDC25 family of protein phosphatases using *vil-Cre-ER^T2^* mice, which facilitate inducible disruption of the *Cdc25s* in epithelial cells but not mesenchymal cells or other cells comprising the stem cell niche. Current approaches to induce cell cycle arrest in the intestine include radiation, chemotherapy or dextran sodium sulfate-treatment. These treatments are suboptimal due to the additional deleterious effects that they impose on the intestinal epithelium such as DNA damage and inflammation.

The response of the small intestine to the loss of epithelial cell proliferation is markedly distinct depending on how cell division is blocked. Chemotherapy and radiation indirectly inhibit cell division by inducing DNA damage. In contrast, blocking Cdc25 function inhibits cell division directly by preventing activation of the cell cycle engine. In the latter case, Wnt-signaling was enhanced, apoptosis was not induced and both crypt number and structure were maintained. Importantly, the inflammatory response was significantly milder in this model. This is in stark contrast to irinotecan treatment, which failed to induce Wnt-signaling and induced an apoptotic response, likely accounting for the severe crypt loss in this model. Furthermore, goblet cells were lost in irinotecan-treated mice and a robust inflammatory response was observed in the intestines of these animals. Another important conclusion that can be drawn from these studies is that the lack of inflammation observed in the small intestines of the *ROSA26*-cre model (*TKO* mice) is not a consequence of functional impairment of inflammatory cells due to CDC25 loss. If this were the case, neutrophil infiltration should have been observed in the intestines of *vTKO* mice where CDC25 loss was restricted to intestinal epithelial cells (Fig. 10I and data not shown for vTKO). These results indicate that the inflammation observed in the small intestines of irinotecan-treated mice is a response to the DNA damage induced by topoisomerase I inhibition.

Despite the development of molecularly targeted therapies, radiation and cytotoxic chemotherapies remain mainstays of cancer treatment. These treatments induce cell cycle arrest and apoptosis of crypt epithelial cells leading to an inflammatory response and in some cases debilitating mucositis in treated patients. The development of novel therapeutic strategies capable of inducing cell cycle arrest in the absence of the accompanying dose-limiting toxicities associated with radiation and chemotherapy would be great advance in cancer treatment. Efforts are currently underway to develop Cdc25 inhibitors for cancer treatment. The cytotoxicity of Cdc25 inhibition needs to be assessed before such inhibitors can proceed to the clinic. The targeted gene knockout studies reported here support the conclusion that the development of therapeutics that impair cell division by inhibiting, but not fully blocking, key regulators of the cell cycle machinery (CDC25s) may have fewer deleterious GI side-effects than chemotherapeutic agents that block cell division by inducing DNA damage. Interestingly CDC25A and CDC25B but not CDC25C are overproduced in several human tumors and their overproduction correlates with poor clinical outcome [23]. Thus, CDC25-inhibitors targeting either CDC25A or Cdc25B may have the useful property of exhibiting anti-tumor activity in those cancers overproducing these phosphatases without inducing the deleterious GI side effects associated with radiation and chemotherapy.

The phenotypes arising in mice deleted for all three family members in intestinal epithelium (*vTKO* mice) were consistent with those observed in mice globally deleted for all three family members (*TKO* mice) using *Rosa-CreER^T^* mice [7]. The phenotype observed in both cases was a profound cessation of proliferation of small intestinal epithelial stem cells and progenitors accompanied by hypoplastic crypts, shortened villi, an overall shortening of small intestinal length and ultimately animal death. These results allow us to conclude that mice globally deleted for the *Cdc25s* die due to the loss of proliferation of intestinal epithelial cells and that the loss of *Cdc25s* in other cell types and tissues of the intestine including mesenchyme, muscle, blood and endothelium does not contribute significantly to the death observed in *TKO* mice [7]. Furthermore, this data demonstrates that enhanced Wnt signaling observed in these mice is a compensatory response to the loss of intestinal epithelial cell proliferation. Finally, this study demonstrates that loss of only two family members (*Cdc25A* and *Cdc25B*) is sufficient to block proliferation of intestinal epithelial cells. Loss of *Cdc25C* alone or in combination with loss of either *Cdc25A* or *Cdc25B* had no effect on intestinal homeostasis or animal viability, even though *Cdc25C* expression is enriched in small intestinal crypts [7]. Thus, CDC25A and CDC25B drive proliferation of intestinal stem and progenitor cells and loss of one can be compensated for by the other.

How then does CDC25C contribute to the cell division cycle? Targeted disruption of *Cdc25C* in mice has failed to reveal unique or compensatory functions for this family member under steady-state conditions. Furthermore, livers from *Cdc25C* knock-out mice regenerate in a manner that is indistinguishable from livers of control mice after a partial hepatectomy (data not shown). Like CDC25A and CDC25B, CDC25C is regulated by protein-protein interactions, intracellular shuttling, proteolysis and reversible phosphorylation [24]. CDC25C has the lowest intrinsic phosphatase activity of the family but is potently activated during mitosis by phosphorylation. Data amassed over the past 20 years support a mitotic function for CDC25C although one study demonstrated that cells deficient in CDC25C arrest at the G1/S-border [25]. Perhaps CDC25C provides a non-essential feed-forward function that serves to accelerate progression from prophase into metaphase and that the effects of its loss cannot be detected within the context of a whole organism.

## Materials and Methods

### Ethics Statement

This study was carried out in strict accordance with the recommendations in the Guide for the Care and Use of Laboratory Animals of the National Institutes of Health. The protocol was approved by the Committee on the Ethics of Animal Experiments of Washington University (Animal Welfare Assurance & Accreditation Number: A33381-01).

### Mice

Generation of mice globally deleted for all three Cdc25s (*TKO*) and methods used to generate and validate *Cdc25B* conditional mice as well as methods used for Quantitative RT-PCR; Southern blotting; histology; Transmission Electron Microscopy; immunohistochemistry; and measurements of GI tract, crypt and villi can be found in Supplemental Material and in Lee et al. [7]. Mice disrupted for *Cdc25A* [7], *Cdc25B* [26]; *Cdc25C* [27] or doubly deleted for *Cdc25B* and *Cdc25C* [28] have been described. All mice were of the strain background C57Bl/6;129X1SvJ. Recombinant DNA research followed the NIH guidelines for research involving recombinant DNA molecules.

### Construction of the *Cdc25B* targeting vector

A 4 kb Bgl II genomic DNA fragment containing exons 1 and 2 of mouse *Cdc25B* was inserted into the Bam HI site of pBluescript SK(+) (Stratagene) to generate pBSK-m25B/B4. Next, an Nhe I genomic fragment of *Cdc25B* containing 7 kb of sequence upstream of exon 1 was inserted into the Nhe I/Xba I site of pBSK-m25B/B4 to generate pBSK-25B/BN. The 5′ homology arm containing exon 1 as well as sequences 5′ and 3′ of exon 1 was isolated as a 3.3 kb fragment by digestion of pBSK-25B/BN with Avr II. PCR was employed to amplify DNA encoding *Cdc25B* from mouse 129/SvJ genomic DNA. The first PCR reaction utilized primers 5′-CTAGTCCTAGGATCACCTCTCCGT-3′ and 5′-GCTGACCACTGACCACAAGG-3′ to amplify 6.9 kb of *Cdc25B* genomic DNA beginning within intron 1 and extending into intron 14. The second PCR reaction utilized primers 5′-CCACCCTAGGCTATCTTTGC-3′ and 5′-CCTAGGAAATGAGACTCATAC-3′ to amplify 4.5 kb of *Cdc25B* genomic DNA beginning within intron 14 and extending to noncoding sequences downstream of exon 16. PCR products were inserted into the Topo TA vector (Invitrogen). p1339 (Genbank Accession # AF335419) was used to assemble the targeting vector. p1339 contains a PGK-NEO cassette (phosphoglycerate kinase promoter driving expression of neomycin phosphotransferase) flanked by 2 loxP sites. p1339 was modified by placing an additional loxP site in Bam HI/Sac I digested p1339 to create p1339(3loxP). The 3.3kb 5′ homologous arm was isolated as an Avr II fragment and cloned into Eco RV digested p1339(3loxP). The 6.9 Kb fragment was isolated as an Spe I/Eco RV fragment and inserted into Bam HI digested p1339(3loxP). The 4.5kb 3′ homologous arm was isolated as an Avr II fragment and cloned into Sal I digested p1339(3loxP).

### Generation of mice harboring the *Cdc25B* mutation

SCC10 ES cells (Siteman Cancer Center at Washington University School of Medicine) were electroporated with linearized targeting vector and selected with Geneticin (G418; Invitrogen) using established protocols developed in the Siteman Cancer Center Murine Embryonic Stem Cell Core (available online http://escore.im.wustl.edu). A total of 142 G418-resistant ES cell clones were analyzed for homologous recombination. Genomic DNA was digested with Hind III and Bam HI followed by Southern blotting with the 5′ and 3′ probes, respectively (shown in Fig. 1A). Four clones were found to be positive by Southern blotting. ES cell clones containing the recombinant allele were expanded and transiently transfected with a plasmid encoding Cre recombinase under the control of the CMV promoter to remove the pGK-NEO cassette. To isolate ES cell clones containing a *Cdc25B* floxed allele and lacking the pGK-NEO cassette, genomic DNA was digested with Kpn I and Southern blotting was performed with the internal probe shown in Fig.1A. ES cell clones that were identified to have a floxed allele were karyotyped and microinjected into C57BL/6 blastocysts, which were subsequently implanted into the uteri of pseudopregnant C57BL/6 X C3HF1 foster mothers. Male chimeras selected by percentage of agouti color were mated to C57BL/6 females. Germ line transmission was determined by agouti coat color. F1 animals were tested for the targeted *Cdc25B* allele by Southern blotting and PCR analysis of tail DNA. PCR analysis was performed with two primers (5′-TGGTCCAGCTGCACTAGAAAG-3′ and 5′-CTTGAGCTTTTGGAGGCTCAC-3′). The sizes of WT and floxed alleles were 383 bp and 433 bp, respectively.

### Quantitative RT-PCR (qRT-PCR)

To determine the efficiency of Cre-mediated recombination at *Cdc25B* locus, total RNA was isolated from the jejunums of tamoxifen-injected animals using Trizol (Invitrogen) and RNeasy (Qiagen). The RNA was reverse-transcribed using Superscript III and random primers (Invitrogen). cDNA from the small intestine of wild type mice was also amplified as a control. cDNA was mixed with Brilliant II SYBR Green QPCR master mix (Stratagene) along with the *Cdc25B* specific primer set (Forward: 5′-TCCAGGGAGAGAAGGTGTCT-3′, Reverse: 5′-TGTCCACAAATCCGTCATCT-3′). Each PCR reaction was performed in duplicate using an MX3005P thermocycler (Stratagene). For normalization, cDNA encoding 18s rRNA was amplified with the primer set (Forward: 5′-CATTCGAACGTCTGCCCTATC-3′, Reverse: 5′-CCTGCTGCCTTCCTTGGA-3′) **and cDNA was diluted at 1∶1000 ratio and used as template.**

### Genotyping by Southern blot analysis and PCR

Mice were euthanized 6 or 7 days post the first tamoxifen-injection, and organs were isolated and lysed overnight in RNE buffer (100 mM NaCl, 10 mM Tris pH 8.0, 25 mM EDTA pH 8.0, 0.5% SDS and 0.1 mg/ml Proteinase K). Genomic DNA was isolated by phenol/chloroform extraction and ethanol precipitation. DNA was digested with Bst XI, separated on an agarose gel and transferred to a nylon membrane. The primers used to make the probes for Southern blotting (Fig. 1A–D) were as follows 5′ Probe: Forward: 5′GTTGTG AGCTGCCATGTGGAT and Reverse: 5′TGCAATCCTACCTTTGTGACG; Internal probe: Forward 5′CGAGTGTGCTCTATGCGACTT and Reverse 5′GTCTTCTCT GGTTTGACTGGT and 3′ Probe: Forward 5′GTAGATAGAACAATG ATCGTCG and Reverse 5′CTTGAGCTTTTGGAGGCTCAC. Primers used for PCR analysis (Fig. 1E) include Forward: 5′TGGTCCAGCTGCACTAGAAAG and Reverse: 5′CTTGAGCTTTTGGAGGCTCAC.

### *vil-Cre-ER^T2^* mice and tamoxifen administration

Mouse lines were maintained in pathogen-free conditions in the animal facility of Washington University School of Medicine. The Animal Studies Committee at Washington University approved all animal procedures. *vil-Cre-ER^T2^* mice have been described [9]. Tamoxifen (Sigma Chemical Co.) was dissolved in sunflower seed oil at a concentration of 10 mg/ml by sonication. Mice were injected IP at 4 mg per 25 g body weight once per day for five consecutive days. Injected mice were monitored daily and sacrificed on day 8 or earlier if they exhibited loss of 20% of original body weight or if they were unable to access food or water. Mice ranged in age from 2 to 3 months at the time of injection.

### X-gal staining

X-gal staining was performed as described by Ahn et al. [29]. Intestines were embedded in Optimal Cutting Temperature Medium (Tissue-Tek). Six µm sections were prepared and fixed for 2 min in 1% formaldehyde, 0.2% glutaraldehyde, 0.02% NP-40, 1 mM NaCl and then incubated in X-gal solution (1 part X-gal dissolved at 40 mg/ml in dimethyl formamide in 40 parts dilution buffer) at 37°C overnight. Dilution buffer consisted of PBS containing 2 mM MgCl_2_, 5 mM potassium ferricyanide, 5 mM potassium ferrocyanide.

### Histology

Mice were euthanized in a CO_2_ chamber. Brain, heart, lung, liver, spleen, kidney and small and large intestines were isolated, washed in phosphate-buffered saline (PBS), fixed in 10% neutrally buffered formalin overnight and embedded in paraffin blocks. 5 µm sections were prepared and stained with H&E or Periodic Acid Schiff (PAS)/alcian blue (AB). A veterinarian in the Division of Comparative Medicine at Washington University in St Louis examined all tissue sections.

### GI tract, crypt and villi measurements

Stomachs as well as small and large intestines, isolated from euthanized mice, were cut open along the cephalocaudal axis, pinned down on a solid plate and fixed overnight with 10% neutrally buffered formalin. Small and large intestines were measured along their entire length and normalized with initial body weights. Villi at the most proximal end of the small intestine were used for length measurements. Cross sections of duodenum were photographed under a dissection microscope and the actual length of villi was calculated by using a scale bar taken in the same picture. 30 villi were measured per mouse.

### Immunohistochemistry

Mouse tissues were fixed in 10% neutrally buffered formalin overnight. Tissue sections were deparaffinized in xylene, rehydrated in a series of alcohols and PBS. Endogenous peroxidase activity was removed by incubating the sections in 3% hydrogen peroxide in methanol for 10 min. Antigen retrieval techniques were performed by boiling the sections in 10 mM sodium citrate (pH 6.0) for 20 min. Sections were blocked with 3% bovine serum albumin, 0.1% Triton X-100 in PBS and incubated at room temperature for 1 h with beta-catenin antibody (BD Transduction lab, 1∶100), cleaved caspase-3 antibody (Cell Signaling, 1∶100) and phospho-Tyr-15 CDK1 antibody (Santa Cruz, 1∶100). MOM kit (BMK-2202, Vector laboratories) was used to block nonspecific binding of secondary antibodies and 3.3′-diaminobenzidine (DAB) was used as a chromogenic substrate. Hematoxylin was used to counter stain. For BrdU single staining, BrdU (Amersham) was IP injected according to the manufacturer's recommendations (2 ml per 100 g body weight). One hour later, mice were euthanized and their intestinal tracts were dissected and processed as described above. BrdU staining was performed with a BrdU staining kit (Zymed) according to the manufacturer's instructions. Only intact well-oriented crypts located half-way between the proximal and distal ends of 2 cm small intestinal sections containing Paneth cells at their base and an intact intestinal lumen composed of a single layer of cells were examined for the presence of nuclear β-catenin.

### TUNEL staining

TUNEL staining was performed according to the recommendations of the manufacturer (Roche) using the In Situ Cell Death Detection Fluorescein kit. Intestinal sections were fixed, deparaffinized and subjected to hydrogen peroxide treatment. Sections were treated with 20 µg/ml proteinase K in 10 mM Tris pH 7.4 for 15 min at 37°C. Sections were then treated with TUNEL reaction mix for 1 h at 37°C. Sections were counterstained with DAPI (nuclei counter stain) using the Prolong Gold mounting media (Invitrogen).

### Transmission Electron Microscopy

Intestines were dissected and fixed in 2% paraformaldehyde/2.5% glutaraldehyde (Polysciences Inc., Warrington, PA) in 100 mM phosphate buffer, pH 7.2 for 1 h at room temperature. Samples were washed in phosphate buffer and post-fixed in 1% osmium tetroxide (Polysciences Inc.) for 1 h. Samples were rinsed extensively in dH_2_0 prior to en bloc staining with 1% aqueous uranyl acetate (Ted Pella Inc., Redding, CA) for 1 h. Following several rinses in dH_2_O, samples were dehydrated in a graded series of ethanol and embedded in Eponate 12 resin (Ted Pella Inc.). Sections of 200 nm were cut with a Leica Ultracut UCT ultramicrotome (Leica Microsystems Inc., Bannockburn, IL) and stained with methylene blue to determine appropriate orientation of the tissue. Sections of 95 nm were then obtained, stained with uranyl acetate and lead citrate, and viewed on a JEOL 1200 EX transmission electron microscope (JEOL USA Inc., Peabody, MA).

### Irinotecan treatment

Irinotecan hydrochloride (CPT-11) was obtained as a 20 mg/ml stock. Prior to administration it was diluted in 0.9% phosphate buffered saline and the mice were injected with either 100 mg/kg or 150 mg/kg intraperitoneally. To analyze effects of irinotecan on intestinal cells at earlier time points, mice were injected with a 100 mg/kg dose at day 1 and day 2 and sacrificed 6 h thereafter. For prolonged exposure studies and to obtain a maximal inhibition of cell proliferation, mice were injected with a dose of 150 mg/kg from day 1 through 6 and sacrificed 6 h following the last dose. One hour prior to sacrifice, mice were also injected with BrdU. Control mice received an equal volume of 0.9% phosphate buffered saline.
